# Prolonged misdiagnosis of mitochondrial encephalomyopathy, lactic acidosis, and stroke-like episodes syndrome: A case report

**DOI:** 10.1097/MD.0000000000036008

**Published:** 2023-11-24

**Authors:** Yun Wang, Weimin Zhang, Xuandong Jiang

**Affiliations:** a Intensive Care Unit, Affiliated Dongyang Hospital of Wenzhou Medical University, Dongyang, Zhejiang, P.R. China.

**Keywords:** epilepsy, hypertrophic cardiomyopathy, MELAS syndrome, misdiagnosis, mitochondrial encephalomyopathy

## Abstract

**Rationale:**

Mitochondrial encephalomyopathy, lactic acidosis, and stroke-like episodes (MELAS) syndrome is a subset of rare mitochondrial diseases characterized by diverse clinical manifestations, which often complicates its diagnosis.

**Patient concerns:**

This report chronicles the experiences of a 14-year-old female patient who underwent multiple misdiagnoses before the eventual identification of MELAS syndrome. Her journey began with symptoms that included growth retardation, hypertrophic cardiomyopathy, and epilepsy.

**Diagnosis:**

The definitive diagnosis of MELAS syndrome was established through genetic confirmation, revealing a mutation in the MT-TL1 gene (m.3242A > G).

**Interventions:**

Upon diagnosis, the patient received targeted symptomatic treatment, which led to pronounced improvements in her symptoms.

**Outcomes:**

The patient’s condition stabilized with the administered treatments, and she exhibited significant symptom relief, emphasizing the importance of accurate diagnosis and timely intervention.

**Lessons:**

This case underscores the imperative for heightened clinical vigilance and thorough differential diagnosis in the face of complex clinical presentations, such as those seen in MELAS syndrome, to ensure timely and appropriate interventions.

## 1. Introduction

Mitochondria are ubiquitous in human cells and serve as primary sites for cellular aerobic respiration, thereby providing essential energy. Genetic defects that cause mitochondrial damage result in mitochondrial dysfunction, leading to impaired adenosine triphosphate synthesis, energy deficiency, and various clinical symptoms. Consequently, mitochondrial diseases can affect all organs in the body, particularly those most reliant on aerobic metabolism, such as the nervous, cardiac, and skeletal muscle systems, and manifest diverse clinical presentations.^[1]^ Mitochondrial diseases are considered rare, with the global prevalence estimated at 1 in 5000; skeletal muscle involvement is termed mitochondrial myopathy, while concurrent brain and skeletal muscle affliction is known as mitochondrial encephalomyopathy. The following clinical phenotypes have been internationally categorized based on the disease manifestations: 1. isolated myopathy, 2. chronic progressive external ophthalmoplegia or Kearns–Sayre syndrome, 3. infantile- and childhood-onset encephalomyopathy, and 4. multisystem disorders accompanied by myopathy. These phenotypes exhibit overlapping features and comprise various subtypes.^[2,3]^ Mitochondrial encephalomyopathy, lactic acidosis, and stroke-like episodes (MELAS) syndrome is among the most common types of mitochondrial encephalomyopathy. Owing to its atypical clinical presentations and diagnostic challenges, MELAS syndrome is frequently misdiagnosed.

This report presents the case of a 14-year-old female patient misdiagnosed with growth retardation, hypertrophic cardiomyopathy, and epilepsy before ultimately being diagnosed with MELAS syndrome. Through this case report, we aim to highlight the importance of differential diagnosis in similar cases to facilitate early and accurate identification of MELAS syndrome.

## 2. Case presentation

A 14-year-old female patient presented to the emergency department on November 28, 2021, complaining of recurrent limb convulsions for more than 24 hours. The patient’s medical history included developmental delay in early childhood and low growth hormone levels. She had received regular growth hormone injections, which were discontinued 3 years before presentation. In August 2019, the patient was hospitalized for dizziness, vomiting, and blurred vision following physical activity and was diagnosed with hypertrophic cardiomyopathy. Metoprolol tartrate (12.5 mg twice daily) was orally administered; however, the patient could not tolerate brisk walking, climbing stairs, or engaging in physical activity. On September 11, 2021, the patient visited our emergency department complaining of a headache, blurred vision, and fever after exercising. Cerebrospinal fluid analysis results were unremarkable, and cranial computed tomography (CT) revealed symmetrical high-density shadows in both basal ganglia regions. After antipyretic treatment, the patient’s vision returned to baseline, and she was discharged. The patient’s family history included hypertrophic cardiomyopathy and diabetes in her maternal grandmother and diabetes in her mother; her younger (aged 6 years) sister had no similar episodes, with normal growth and development, although she had a mitochondrial gene mutation similar to that in the patient (mutation rate, 69%).

**Figure 1.**
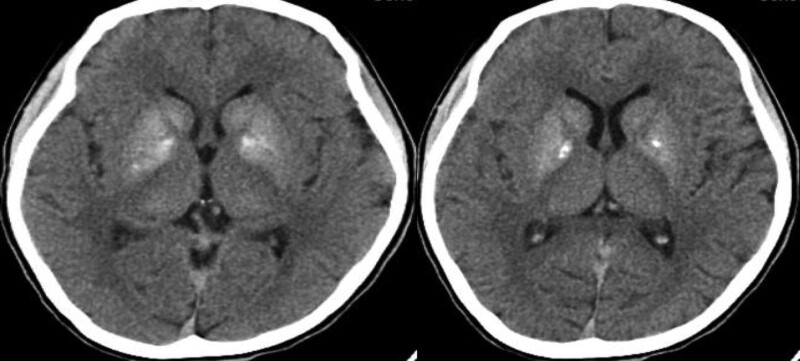
Computed tomographic scan demonstrating symmetrical high-density shadows in both basal ganglia regions.

**Figure 2.**
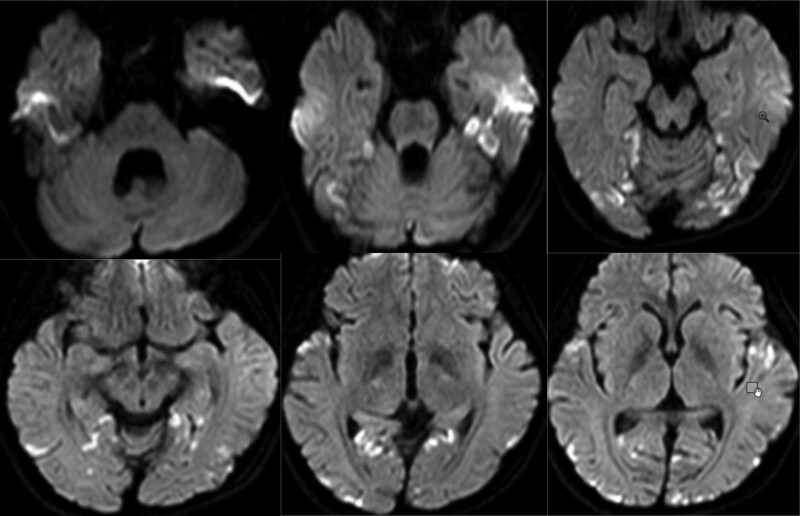
Magnetic resonance imaging presents abnormal signals in bilateral temporal lobes, hippocampi, occipital lobes, and frontoparietal regions, with high signal intensity in the basal ganglia in T1-weighted imaging.

**Figure 3.**
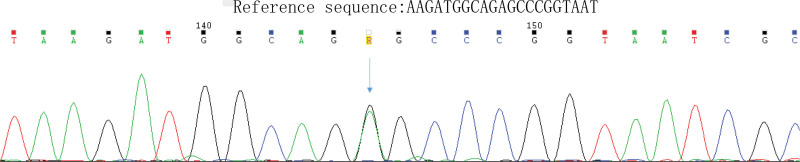
Sanger sequencing validation diagram of the patient.

On arrival at the emergency department, the patient’s vitals were recorded as follows: heart rate, blood pressure, respiration rate, oxygen saturation level, and body temperature of 110 beats/min, 107/75 mm Hg, 25 breaths/min, 96%, and 38 °C, respectively. She was confused and experienced intermittent limb convulsions. Hypoxia was observed, and endotracheal intubation and mechanical ventilation were initiated. Arterial blood gas analysis revealed pH, partial pressure of carbon dioxide, partial pressure of oxygen, and lactate levels of 6.879 (normal range 7.35–7.45), 53.8 mm Hg (normal range 35–45 mm Hg), 107.0 mm Hg (normal range 80–100 mm Hg), and 19.0 mmol/L (normal range 0.5–1.6 mmol/L), respectively. Complete blood count analysis revealed white blood cell count and neutrophil level of 28.79 × 10^9^/L (normal range 3.5–9.9 × 10^9^/L) and 0.86 (normal range 0.4–0.75), respectively. Brain CT findings were consistent with symmetrical high-density shadows in both basal ganglia (Fig. 1). The patient was diagnosed with the following: 1. status epilepticus, 2. suspected mitochondrial encephalomyopathy, and 3. hypertrophic cardiomyopathy. The patient was admitted to the intensive care unit for treatment, which included levetiracetam (0.5 g twice daily) orally administered with perampanel (4 mg daily) for seizure control; mannitol (100 mL intravenously administered twice daily) for dehydration and to reduce intracranial pressure; hypothermic brain protection; coenzyme Q10 (10 mg orally administered 3 times daily); vitamin B1 (10 mg orally administered twice daily); vitamin B2 (5 mg orally administered twice daily); and vitamin B6 (50 mg intravenously administered daily). Cranial magnetic resonance imaging (MRI) revealed abnormal signals in the bilateral temporal lobes, hippocampi, occipital lobes, and frontoparietal regions, with high signal intensities in the basal ganglia on T1-weighted imaging (Fig. 2). Following a departmental discussion, mitochondrial encephalomyopathy was suspected, and whole mitochondrial genome sequencing was performed externally (Dean Medical Laboratory Center, Hangzhou, China). Genetic test results (Fig. 3) showed high-throughput sequencing, and analysis of the entire mitochondrial genome (37 genes) and the MT-DLOOP region revealed one pathogenic mutation in *MT-TL1* (m.3242A > G; mutation rate, 49%), thus confirming the diagnosis of MELAS syndrome. Given the absence of definitive treatment, we used a supportive care regimen. The patient was weaned off mechanical ventilation on hospital day 13, transferred to the general ward on day 17, and discharged on day 25. Throughout this period, no adverse or unexpected incidents were reported.

### 2.1. Follow-up

Follow-up via telephone 6 months later revealed that the patient was regularly taking antiepileptic drugs and metoprolol tartrate, had stable disease control, and could tolerate daily activities. One year later, she was admitted to our hospital for another convulsion of the right lower limb, and her antiepileptic drugs were changed to levetiracetam tablets 0.75 g bid and lacoxamide tablets 50 mg bid. Her condition improved, and she was discharged.

Written informed consent was obtained from the legal guardian of the patient.

## 3. Discussion

MELAS syndrome is a complex and diverse multisystem disorder caused by mitochondrial DNA mutations that are frequently misdiagnosed or overlooked.^[3]^ Our patient had an atypical family history and symptoms, including recurrent limb convulsions, dizziness, vomiting, and vision decline. The patient was initially diagnosed with growth retardation, hypertrophic cardiomyopathy, and status epilepticus. However, genetic sequencing ultimately confirmed the diagnosis of MELAS syndrome.

The pathogenesis of MELAS syndrome primarily involves mitochondrial DNA mutations. *MT-TL1* gene mutations lead to mitochondrial dysfunction, thereby affecting energy metabolism and redox balance.^[4]^ MELAS syndrome typically manifests during childhood following normal early development, with recurrent-relapsing episodes characterized by epileptic seizures or dementia. The diagnosis is usually based on lactic acidosis or ragged-red fibers identified in skeletal muscle biopsies. However, genetic advancements have facilitated the identification of causative mutations through genetic testing without relying on muscle biopsy results.^[5]^ Our case particularly demonstrates pathogenic mutation in *MT-TL1* (m.3242A > G; mutation rate, 49%) through mitochondrial whole-genome sequencing, which validates the MELAS syndrome diagnosis and circumvents the inconvenience and risks associated with reliance on muscle biopsy findings.

Cardiac involvement is a common manifestation in patients with mitochondrial myopathies; however, the incidence of cardiac disease in patients with MELAS syndrome is relatively low.^[6]^ The patient and her grandmother had hypertrophic cardiomyopathy, similar to that of a 27-year-old woman with MELAS syndrome and diminished exercise capacity, as revealed by echocardiography.^[7]^ Cardiovascular MRI can reveal the characteristics of myocardial injury in patients with mitochondrial myopathies.^[8]^

A salient feature of our case management involved the collection of in-depth patient history and comprehensive physical examination, paired with appropriate auxiliary tests, including genetic analyses, which obviated further misdiagnosis or missed diagnosis of MELAS syndrome. Particularly, the role of MRI scans is crucial. In contrast to typical embolic or thrombotic ischemic stroke, brain diffusion-weighted MRI shows high signals that do not follow a vascular distribution. The apparent diffusion coefficient on MRI may not necessarily decrease (as it does during tissue infarction) and may even increase or exhibit mixed patterns. Acute MRI signal changes are not static and may shift, fluctuate, or recede.^[9,10]^ In our case, both CT and MRI revealed typical findings, which aided in the definitive diagnosis.

Nevertheless, there are limitations. Notably, no specific therapeutic or curative measures exist for MELAS syndrome, and internationally recognized treatments may only improve symptoms without affecting disease progression. Current therapeutic measures include antiepileptic drugs, coenzyme Q10 as a mitochondrial nutrient, and targeted treatments for comorbidities; however, future gene therapies show promising results.^[11,12]^

## 4. Conclusions

This case emphasizes the importance of increasing awareness of MELAS syndrome among clinicians to promote early and accurate diagnosis of this disease. Treatment for MELAS syndrome primarily involves symptomatic relief, and proactive interventions can help improve patient prognosis.

## Acknowledgments

We would like to thank Editage (www.editage.cn) for English language editing.

## Author contributions

**Conceptualization:** Weimin Zhang.

**Investigation:** Yun Wang.

**Resources:** Yun Wang.

**Visualization:** Xuandong Jiang.

**Writing – original draft:** Yun Wang.

**Writing – review & editing:** Xuandong Jiang.
